# Exploring the Universe of Protein Structures beyond the Protein Data Bank

**DOI:** 10.1371/journal.pcbi.1000957

**Published:** 2010-11-04

**Authors:** Pilar Cossio, Antonio Trovato, Fabio Pietrucci, Flavio Seno, Amos Maritan, Alessandro Laio

**Affiliations:** 1 International School for Advanced Studies (SISSA-ISAS) and CNR-INFM DEMOCRITOS, Trieste, Italy; 2 Universitá degli Studi di Padova and CNISM, Unitá di Padova, Padova, Italy; 3 Centre Européen de Calcul Atomique et Moléculaire (CECAM), Ecole Polytechnique Fédérale de Lausanne (EPFL), Lausanne, Switzerland; Peking University, China

## Abstract

It is currently believed that the atlas of existing protein structures is faithfully represented in the Protein Data Bank. However, whether this atlas covers the full universe of all possible protein structures is still a highly debated issue. By using a sophisticated numerical approach, we performed an exhaustive exploration of the conformational space of a 60 amino acid polypeptide chain described with an accurate all-atom interaction potential. We generated a database of around 30,000 compact folds with at least 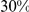 of secondary structure corresponding to local minima of the potential energy. This ensemble plausibly represents the universe of protein folds of similar length; indeed, all the known folds are represented in the set with good accuracy. However, we discover that the known folds form a rather small subset, which *cannot* be reproduced by choosing random structures in the database. Rather, *natural* and *possible* folds differ by the contact order, on average significantly smaller in the former. This suggests the presence of an evolutionary bias, possibly related to kinetic accessibility, towards structures with shorter loops between contacting residues. Beside their conceptual relevance, the new structures open a range of practical applications such as the development of accurate structure prediction strategies, the optimization of force fields, and the identification and design of novel folds.

## Author Summary

Protein structure and biological function are determined by their sequence, but proteins of different sequence or function can share the same structure. To rationalize this puzzling observation we explored by computer simulations the universe of all possible folds for proteins of relatively small length. We find that nature exploits a relatively small corner of this universe. Evolution selected this region under the guidance of a simple principle: reducing the entanglement in the bundle formed by the protein in its folded state. This makes bundles with shorter loops preferable. The set of structures that we make available will open a range of practical applications in biomedical sciences.

## Introduction

The total number of distinct protein folds which have been experimentally solved is very small compared to the amount of genome-wide protein sequences [1], [2]. Indeed, folds are evolutionarily more conserved than sequences and the same fold can house proteins performing different biological functions [3], [4]. Thus a fundamental question concerns the extension of the library of protein folds: are the observed structures a small fraction of the whole fold universe? If so, then is it because evolution has not yet run enough to explore it or rather because a selection principle is on which has slowed down/stopped the search for alternatives?

Addressing these issues on the basis of the principles of physics and chemistry is a question of fundamental importance, currently at the center of intense investigation. Several properties of the folding process have been shown to depend more on the fold topology than on the specificity of the aminoacids [5]–[10]. For a few proteins, native backbone geometries were shown to be closely mimicked by local energy minima of poly-alanine chains [11]. More recently, a unified approach to the origin of protein folds was proposed in which the inherent anisotropy of a chain molecule, the geometrical and energetic constraints placed by hydrogen bonds, steric hindrance and hydrophobicity yield a free energy landscape with minima resembling protein structures [12]–[14]. One of the predictions is that a limited library of folds exists. Along the same lines, based on a coarse grained model, Zhang *e*t al proposed [15] that there is a one-to-one correspondence between the Protein Data Bank (PDB) library and the structures that one can obtain with a homopolymer from the requirement of “having compact arrangements of hydrogen-bonded, secondary structure elements and nothing more” [15]. A different scenario has been proposed in ref. [16] where, by using structure prediction method based on an idealized secondary structure lattice representation they argued that the space of possible folds might be larger than the space of natural folds.

Recent advances in supercomputing power and sampling methods [17], [18] allow us addressing these issues by accurate atomistic simulations. We here describe the results of a 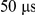 molecular dynamics simulation of a 60 amino acids polypeptide chain performed with an accurate all-atom interaction potential and a setup specifically designed in order to extensively explore the configuration space. The length of 60 was chosen because it represents the limit of what can be simulated with our computational resources. Natural proteins are on average much longer than 60 amino acid, but several autonomously folded domains of this size exist [19], making the comparison between simulation and nature meaningful. In the simulation we visit practically all the 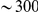 folds observed in nature for proteins of comparable length. However, at variance with what found in [15], we find that natural folds are only a small fraction of the structures that are explored. Many of the structures found in our simulation resemble real proteins (in terms of secondary content, stability and compactness) but have not been observed in nature. This finding immediately rises a question on the nature and meaning of these novel folds: why are they not exploited in real proteins? Do natural folds have something “special” or have they simply been selected randomly?

## Results

### A library of 30,000 folds

**Figure 1:**
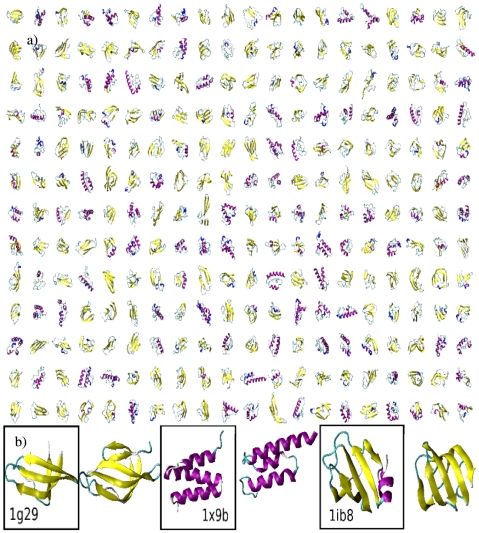
Gallery of representative VAL60 structures generated by molecular dynamics. (a): A selection of 260 out of the 30,000 structures generated by MD, visualized by VMD [39]. The structures were selected from the 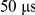 molecular dynamics trajectory if they satisfied the following conditions: (i) have more than 30% of secondary content according to DSSP [37] (ii) have a gyration radius smaller than 15 Å; (iii) be separated more than 50 ps in simulation time. The structures obtained in this manner are further optimized by steepest decent with 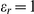 until a local potential energy minimum is reached (see Methods). (b): Examples of successful alignments. The CATH structure is represented together with its VAL60 equivalent for three cases.

By using a state-of-the art enhanced sampling technique [18], we simulate a 60 amino acid polyvaline (VAL60) described by an all-atom potential energy function [20] as explained in Methods. This allows generating, in 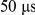 of simulation, 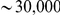 structures characterized by a significant secondary content and a small radius of gyration. A movie with a short part of the trajectory (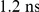) is available as Video S1. It shows the exploration proceeds mostly by local reorganization of secondary structure elements. From time to time the system unfolds completely, exploring a totally independent topology. A selection of the 30,000 structures is represented in Fig. 1-a and a repository, with their all-atom configuration, is available at http://dx.doi.org/10.5061/dryad.1922. By steepest descent optimization (see Methods) we verified that even if these structures have been obtained with an enhanced sampling technique, they closely correspond to local minima of the potential energy surface of VAL60. Consistently with Ref. [11], they also correspond closely to local minima of the potential energy surface of polyalanine (ALA60) (see Methods).

**Figure 2:**
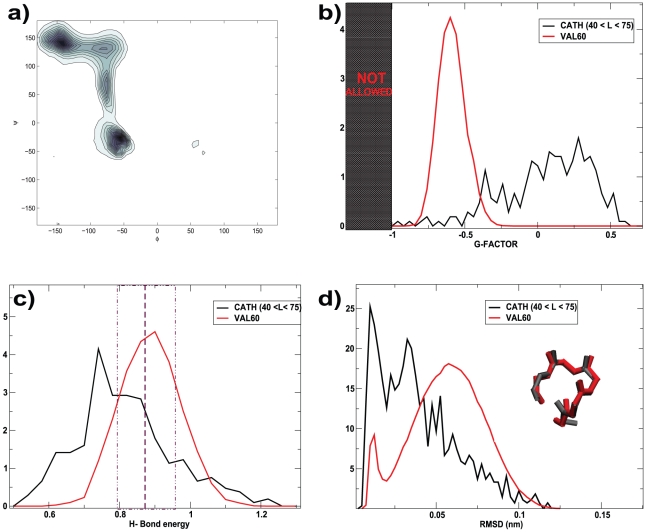
Structural quality assessment for the VAL60 set. (a) Ramachandran plot for the VAL60 structures. (b) G-factor [21] distribution and (c) H-bond energy distributions [21] for the VAL60 and CATH (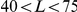) sets. (d) Minimum RMSD distribution for a set of 150000 5 amino acids long fragments of the VAL60 set, and 1000 fragments of the CATH set. Inset: an example of an alignment between two fragments with a RMSD of 0.7 Å.

Even though these structures correspond to local minima, one still wonders if their structural quality is good and if they resemble real proteins. In order to address this issue, we monitored several structural quantities on our dataset. In Fig. 2-a we show the Ramachandran plot of the VAL60 structures. One can see that the dihedrals populate the allowed regions. The relative height of the various peaks is determined by the probability to observe the different secondary structural elements and the random coil in the full dataset. The “stereochemical quality” of the VAL60 set was also assessed using PROCHECK [21]. This program provides an overall quality measure, called G-factor, which takes into account dihedrals, bond lengths and angles, as compared with stereochemical parameters derived from well-refined, high-resolution structures. If the G-factor is higher than −1.0 the structure is considered to be “normal”. In Fig. 2-b the G-factor distribution is shown for the VAL60. For a comparison, we computed the same distribution also for the structures of length smaller than 75 amino acids belonging to the CATH database [19]. We also used PROCHECK to estimate the average hydrogen bond energy. The distributions of this quantity for VAL60 and CATH is shown in Fig. 2-c and compared (dash line) with its ideal mean and standard deviation [21]. For the VAL60 set the G-factor and the H-bond energy, though not as good as for CATH, are in accordance with what is expected for realistic proteins. Lastly, in order to check if medium size structures generated by our sampling procedure are representative of the PDB, the VAL60 structures were fragmented in small 5 amino acids long structures and were compared by backbone RMSD [22] to all the fragments of the same length found in CATH. The minimum RMSD value was obtained for each small fragment. The distribution of this quantity is shown in Fig. 2-d. It is found that the VAL60 fragments have on average at least one CATH structure within 0.6 Å of RMSD. For all the structural descriptors we considered the VAL60 distributions are similar but not identical to the ones of real proteins, due to the fact that in our simulation we considered an homopolymer formed by only one amino acid, valine. Taken together the data shown in Fig. 2 demonstrate our first major result: finding by molecular dynamics at an all-atom level a library of 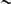
30000 protein-like structures.

### All the known folds between 40 a.a. and 75 a.a. are reproduced

**Figure 3:**
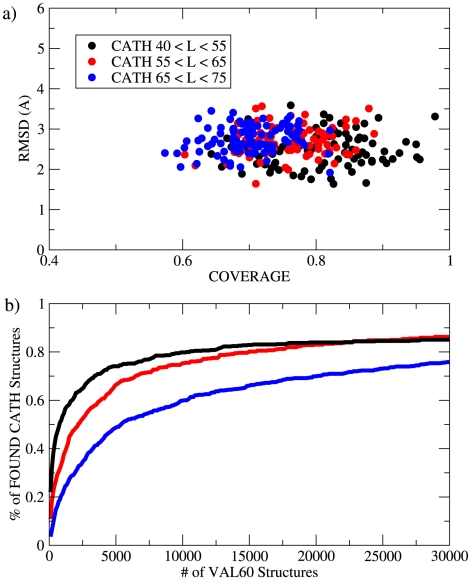
Similarity between the VAL60 set and the PDB structures from the CATH database. (a) Coverage vs RMSD (see Methods) for the CATH proteins divided in different length classes with respect to their most similar VAL60 structure. (b) Percentage of CATH structures that are reproduced by a structure in the VAL60 set (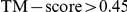) as a function of the number of the VAL60 structures obtained in the simulation.

The VAL60 structures obtained in this manner, at a first sight, cannot be distinguished from folds adopted by proteins. In order to understand how many independent structures are actually explored, and if the set contains all the known folds, a measure of the degree of similarity between two protein structures is needed. We used the TM-align approach [23], which gives, as three quantitative outputs, the coverage, the root mean square distance (RMSD) between the aligned residues, and the TM-score (see Methods). Following Ref. [15], we first checked if the set of structures generated by molecular dynamics reproduces all the known folds. As a target set we here considered the CATH database [19], that is successfully used in structural studies to classify protein folds. Other choices were also considered (see Text S1). For each structure in the CATH database, we searched, in the set of the 30,000 structures of VAL60 generated by molecular dynamics, for its most similar structure as quantified by the TM-score. In Fig. 1-b, three CATH structures with their respective VAL60 equivalent are shown. As shown in Fig. 3-a, for almost every CATH structure it is possible to find a VAL60 structure that is very similar. For CATH structures of length between 55 and 65 amino acids the average coverage is 75%, and the average RMSD is of only 2.8 Å. The VAL60 set reproduces, with even greater success, CATH structures of shorter length. Instead, structures of 65 or more amino acids are reproduced less accurately, as the maximum coverage that can be attained is, by definition, smaller than their length. However, even in these cases, the RMSD restricted to the aligned residues is small, of 3 Å or less. Comparison of the VAL60 set with even longer chains is not considered here: the long chains can contain extra secondary structure elements that do not significantly affect the quality of the alignment but change the topological details of the fold.

The excellent capability of the VAL60 set of reproducing the known folds is confirmed by monitoring the progress of exploration as a function of the number of structures found during the simulation. At this purpose, we assumed that a CATH structure is “found” when molecular dynamics explores a VAL60 structure whose TM-score (with respect to the CATH structure) is higher than 0.45. Visual inspection reveals that two structures of similar length and of relative TM-score larger than 0.45 are structurally and topologically similar. In Fig. 3-b we plot, for different length classes, the fraction of CATH structures that are found as a function of the number of VAL60 structures (which is approximately proportional to simulation time). At the end of the simulation, for length L = 55–65 the fraction of found structures is 86% (85% for L = 40–55 and 78% for L = 65–75). 100% of the structures of length L = 40–65 are reproduced within a TM-score of 0.4. This shows that the computational setup used in this work allows us to explore the majority of the folds in nature, at least within the limited range of lengths considered. This is the second main message of our study and confirms the results of Ref. [15] obtained with a simpler potential energy function.

### The universe of *possible* folds is much larger than the PDB

The exploration of VAL60 structures by molecular dynamics proceeds in an almost random manner, with no obvious preference for a specific class of folds or secondary structure element. Indeed we checked that it is, on average, equally likely to find a specific CATH structure as finding a VAL60 structure for the second time (see Methods). In other words, in our sampling strategy there is no particular bias for generating a structure observed in nature. However, one realizes that the two sets of structures, CATH and VAL60, cannot be fully equivalent. Indeed, according to a clustering procedure (see Methods), in 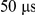 the simulation explores 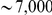 independent structures, much more than the structures in CATH (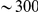 in a length range between 40 and 75).

**Figure 4:**
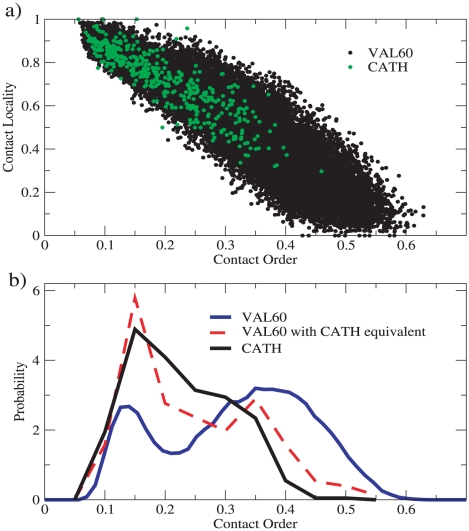
Contact order and Contact Locality distributions of CATH and VAL60. (a) CO vs CL (see Methods) represented for the CATH set of length 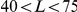, and the VAL60 set. (b) CO distributions for the CATH set of length 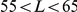, VAL60 set and for the subset of independent VAL60 structures that have 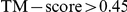 with a structure in the same CATH set. Independent structures are obtained as described in Methods.

One could argue that finding or not a one-to-one correspondence might just depend on the chosen similarity threshold [24]. In order to quantitatively investigate this issue, we addressed the following question: Do structural descriptors exist whose distributions are different between the two sets CATH and VAL60? If the answer is yes, a biased search mechanism reflecting an evolutionary pressure may be envisaged. Otherwise a random search mechanism in a continuous structure space may be enough to account for the choice of the observed folds out of all possible structures. While at first sight structures belonging to the VAL60 and CATH sets look indistinguishable, a more detailed analysis reveals that several VAL60 structures include a large fraction of parallel 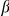-sheets. This secondary structure element is much less common in the CATH set restricted to 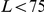. We quantify this observation by looking at the distributions of normalized contact order (CO) and the contact locality (CL) (see Methods). The distribution of CATH is significantly restricted towards lower CO and higher CL values with respect to VAL60 (see Fig. 4-a), consistent with the observation that parallel 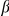-sheets are found less frequently in CATH. We have checked that this discrepancy is not due to the specific simulation setup (see Methods).

We also checked that the CO distribution computed for the subset of VAL60 that are recognized to be similar to CATH is largely overlapping with the CO distribution for the CATH set (see Fig. 4-b). This demonstrates the consistency of the similarity measure provided by the TM-score. We also analyzed the distribution of the CO restricted to the different structural classes. The bias towards low CO is not effective for all-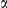 structures (see Fig. S6), whereas is active for all-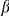 and 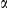-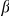 structures. All these results suggest that, among all possible conformations physically attainable by polypeptide chains, real protein structures were selected under a bias towards low CO. This is the third main message of our study: As observed with the coarse grained model of ref. [16], there is no one-to-one correspondence between the PDB library and the ensemble of compact structures with significant secondary content.

## Discussion

By using atomistic simulations and a powerful enhanced sampling technique we have generated a database of 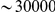 structures corresponding to energy minima of a 60 amino acids polypeptide. Clearly, the length of 60 amino acids used in the simulation does not provide a complete representation of the full protein universe, which includes a very large amount of much longer proteins. However, our results indicate that, within the limited length range we considered, the VAL60 set is indeed representative of the space inhabited by real proteins. In fact, this set includes all the folds existing in nature for proteins of similar size, confirming that the observed protein folds are selected based on geometry and symmetry and not on the chemistry of the aminoacid sequence [5]–[15]. However, we find that the known folds form only a small fraction of the full database. Natural folds are indistinguishable in terms of secondary content and compactness from non-natural folds, but are characterized by a relatively small contact order and a relatively high contact locality. Why has nature made this choice? One can argue that, due to a higher 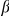-structure content, large CO structure could have a higher tendency to aggregate. Another possible explanation relies on kinetic accessibility, as the contact order is known to correlate with the folding time of two-state globular proteins [25]. Evolution might have selected the folds under the guidance of a simple principle: reducing the entanglement in the bundle formed by the protein in its folded state. Bundles with shorter loops might be preferable, as they are explored more easily starting from a random coil.

**Figure 5:**
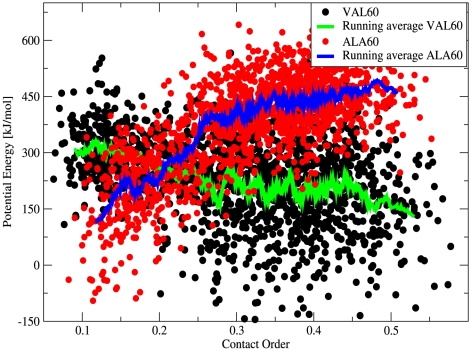
Correlation between potential energy and contact order for VAL60 and ALA60 structures. For a subset of 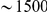 structures from the VAL60 set we generated a corresponding set of ALA60 structures by finding the local potential energy minima after conversion of valine into alanine residues (see Methods). We then sorted all the structures according to their CO. Each point in the figure corresponds to a structure. We also represent the running average of the energy over a window of 50 structures.

How has nature been able to select low contact order structures? In order to address this issue, we investigated the role of specific amino acids in selecting a fold among the possible structures. At this scope, we compared the correlation between potential energy and CO of the structures obtained by energy minimization of VAL60 and ALA60 (see Methods). Fig. 5 vividly demonstrates that different low energy structures may be discriminated when different sequences are mounted on all the possible “presculpted” structures [12]. Whereas energetically VAL60 prefers structures with high CO and a large content of strands, ALA60 promotes conformations with low CO and which are rich in helices. Evolution, possibly also guided by the kinetic bias hypothesized above, can then proceed by using a repertoire of 20 types of amino acids, to select and design the sequences which minimize the free energy of a desired structure against other competing structures.

As a final remark, we believe that the VAL60 structures and the computational procedure to generate them, also with different types of amino acids and with different lengths, may play a key role in future developments. The availability of a rich library of possible folds and realistic decoys could allow for major advances in the two main applicative challenges in protein physics: the prediction of the native state of any given sequence and the design of the sequence folding into a desired fold. They might be also used to check predictions in synthetic biology [26], [27]. Furthermore the library could be exploited to obtain models of misfolded protein structures related to neurodegenerative diseases [28]. We have shown that generating a huge set of realistic structures is feasible with a computational analysis based only on ab-initio physico-chemical information, with no need of using knowledge-based potentials as in state-of-the-art approaches to protein structure prediction and design [29].

## Materials and Methods

### Setup of the simulation

Molecular dynamics (MD) simulations are performed using the AMBER03 [20] force field and the molecular dynamics package GROMACS [30]. Simulations are mainly performed in vacuum, but tests have been performed also in water solution (see below). The temperature is controlled by the Nose-Hoover thermostat, and the integration time step is 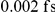. In order to explore the conformational space we use bias-exchange metadynamics (BE-META) [18], [31] with 6 replicas. BE-META is a combination of replica exchange [32] and metadynamics [33], in which multiple metadynamics simulations are performed at the same temperature. Each replica of the system is biased with a one-dimensional metadynamics potential acting on a single collective variable (CV). The CVs are described in detail in [34] and are designed in order to evaluate by a differentiable function of the coordinates the fraction of a secondary structure element (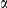-helix, parallel 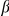-sheet and antiparallel 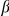-sheet). For instance, for the antiparallel 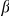-sheet the variable counts how many pairs of 3-residue fragments in a given protein structure adopt the correct 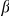-conformation, measured by the RMSD from an ideal block of antiparallel 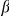 formed by a pair of three residues. We use six CVs: 3 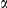-CVs each biasing one third of the protein, 1 anti-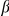 CV, and 2 para-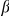 CV. The Gaussians entering in the metadynamics potential are added every 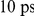. Their height and width are 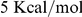 and 0.3. Exchanges between the biasing potentials are allowed every 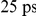. The exchanges greatly enhance the capability of the dynamics of exploring new structures [18], [35]. These parameters have been optimized according to the criteria of Ref. [36].

The main scope of this work is exploring exhaustively the conformational space of an average length polypeptide described by a realistic potential energy function. The final choice of simulating VAL60 in vacuum with 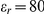 at 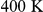, and then optimizing the configurations with 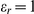 was taken after considering several alternatives. We first considered performing the simulation on a 60-alanine in vacuum (ALA60), as alanine is used in Ref. [11]. This system was evolved using the BE-META setup described above for 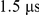 generating 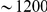 structures with a high secondary content. However, the structures generated in this manner are too compact to be comparable with experimental structures. Indeed, the histogram of the radius of gyration for ALA60 is peaked approximately 1 Å too low with respect to what observed for real proteins of similar length (see Fig. S1). This is due to the relatively low steric hindrance of the side chain of ALA. The same histogram computed for VAL60 is instead fully consistent with the distribution observed in real proteins. We also performed test simulations of VAL60 solvated in TIP3P water at 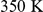. This system was evolved for 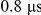 with the same BE-META setup. In this case 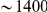 structures with a high secondary content are found, but most of these structures are not independent, as the correlation time in water is much larger than in vacuum. More importantly, the structures generated in water have on average a large radius of gyration (see Fig. S1). This is an indication that at 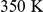 the system explores mainly non-compact structures. Of course, one could perform the simulation at lower temperature, but this would lead to an even larger correlation time, making an exhaustive exploration of the configuration space too time consuming with existing computational resources. Performing the exploration with 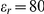 is not strictly necessary, as test simulations performed with 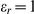 are also able to explore structures with a high secondary content. However, VAL60 with 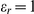 has a relatively high preference for 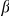 structures (see Fig. 5). With 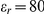 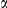 and 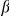 structures become approximately isoenergetic for VAL60, removing a possible bias in the exploration (see also Fig. S5).

### VAL60 and ALA60 minimization

The VAL60 set was generated by molecular dynamics in vacuum at 400 K, biasing the system by metadynamics potentials aimed at producing secondary structure elements. One wonders if the structures that are explored in this manner have protein-like topologies only because of the bias, and would fall apart in normal conditions. In order to address this issue, for all the structures generated by molecular dynamics we performed a steepest decent (SD) simulation with 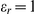, aimed at localizing the closest potential energy surface minimum. For the last configuration the 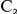 RMSD was calculated with respect to the initial structure. The distribution of this quantity is shown in Fig. S2. Most of the structures do not drift significantly apart from the initial configuration, and the 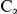 RMSD remains relatively small, within 2 Å in most cases. Thus, we conclude that the VAL60 structures generated by molecular dynamics are close to local energy minima. The set of structures generated in this manner form the database on which we perform the analysis.

We also checked if the structures that are generated in this manner are stable if the homopolymer chain is formed by another amino acid. At this purpose, 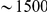 VAL60 structures were chosen randomly. For each of these structure the valines were replaced by alanines (ALA60). Following the same procedure described above, a SD simulation was run until the closest local minimum is reached. The 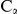 RMSD from the initial ALA60 configuration was calculated. The distribution of this quantity is shown in Fig. S2. Quite remarkably, even if one changes the amino acid sequence from VAL60 to ALA60 the structures do not change significantly, remaining within 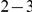 Å of 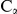 RMSD from the initial structure. This confirms the prediction of Ref. [11].

### TM-align algorithm

The similarity between two different structures is assessed using the TM-align algorithm [23]. This method, regardless of the primary sequence of the two proteins, attempts to align their secondary structure elements allowing insertions and deletions of residues. The fraction of aligned residues is called *coverage*, and is the first measure of similarity. Afterward, the algorithm finds the rotation and translation that minimizes the relative distance between pairs of aligned residues (*RMSD*). The optimal coverage and RMSD are then combined into a single similarity measure, the *TM-score*. The original version of the TM-align algorithm has been modified in order to assign the secondary structure elements with more accuracy. Instead of considering only the 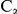 coordinates as in Ref. [23], our modified version reads for each protein the secondary structure assignment given by DSSP [37]. When the proteins have different lengths, the length of the target protein is used in the TM-score definition [23]. The TM-score is equal to one for two identical structures. Two structures are considered to represent the same fold if their TM-score is greater than 0.45, while for two randomly chosen structures the TM-score is approximately equal to 0.25.

### Finding the independent structures

In order to find the independent structures we proceeded as follows: first we selected the structure with the largest number of neighbors, namely with the largest number of structures at a TM-score larger than 0.45. We assign it as the first independent structure and remove it, together with all its neighbors, from the list of structures. We iterate this procedure until the list is empty. In Fig. S3 we plot the number of independent structures found as a function of the number of structures explored by MD. This data can be accurately reproduced with a double exponential fit (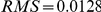), which allows estimating as 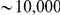 the number of independent structures that would be explored in an infinitely long MD run.

### CATH and VAL60 structures are explored with equal probability

We consider a small fraction of the MD trajectory used for generating the VAL60 dataset. In this fraction of the trajectory 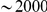 independent structures are generated. Using the rest of the trajectory, we compute the number of times 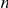 that each of these structures is observed (namely, the number of times a structure with relative TM-score larger than 0.45 is visited). The histogram of 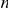 is calculated for 20 different sets, each including 100 VAL60 structures. Its average and standard deviation (error bars) are plotted in Fig. S4. This is compared to the same histogram computed for the CATH set with 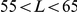 (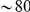 structures). Strikingly, the two histograms are very similar, indicating that the probability of finding a CATH structure in this length range is similar to the probability of finding a VAL60 structure a second time.

### Contact order and contact locality

Two residues are considered to be in contact when at least one pair of their heavy atoms is found at a distance smaller than 3.5 Å. The contact order (CO) [25] is defined as the average sequence separation between contacting residues divided by the chain length. The contact locality (CL), is a structural descriptor that counts the fraction of contacting residue pairs which are formed within the same half of the chain [38]. The total number 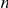 of pairwise contacts is 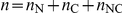, where 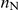 and 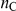 are the contacts between residues both belonging to the half of the chain towards the N-terminus and the C-terminus, respectively, and 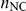 are the contacts between residues belonging to different halves of the chain. CL is then defined as 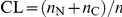.

### VAL60 structures with high or low CO are explored with approximately equal probability

One of the main results described in the work is that, on average, the VAL60 structures have higher CO than CATH structures. In order to find out if the biasing procedure favors high CO structures we separate the 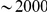 VAL60 structures in two classes: low CO (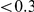) and large CO (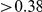), and we calculate the probability to find a structure 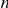 times in the simulation (same procedure as above). The two distributions with the respective error bars are shown in Fig. S5. From the graph, it can be concluded that the two distributions are similar but it is marginally easier for VAL60 to re-generate more times low CO structures rather than high CO ones. Thus, the VAL60 system is able to sample low CO structures with a marginally higher efficiency. This is possibly due to the fact that low CO structures are kinetically encountered more often in a random search guided only by a bias towards high secondary structure content. This allows concluding that the large number of high CO structures that is obtained by molecular dynamics is not due to a bias in the sampling procedure.

### Contact order for different secondary structure classes

The results found in Fig. 4 show that there is a bias towards low CO structures for the CATH set. In order to find out how this bias acts for different structural classes, the CO distributions was calculated for all-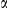 structures and all-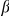 structures of CATH and VAL60. The results are shown in Fig. S6. While the bias towards low CO is present for all-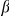 structures, for all-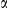 structures it is not effective. It is also remarkable that the CO distribution for 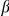 structures in the VAL60 set that are similar to a CATH structure is very similar to the probability distribution for the all-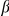 CATH structures.

## Supporting Information

Figure S1 Distribution of the radius of gyration for the VAL60, VAL60+WATER,ALA60 and CATH 55–65 sets of structures.
(0.02 MB EPS)

Figure S2 Cα RMSD distributions for the 30,000 VAL60 and the 1500 ALA60 minimized through SD. The RMSD is measured with respect to the initial configuration.
(0.02 MB EPS)

Figure S3 Number of independent VAL60 structures as a function of the number of structures obtained in the MD trajectory.
(0.02 MB EPS)

Figure S4 Probability of finding *n* times a CATH structure and a VAL60 structure.
(0.02 MB EPS)

Figure S5 Probability of finding a structure in the VAL60 trajectory for different CO classes.
(0.02 MB EPS)

Figure S6 Contact order for different structural classes. The CATH and VAL60 sets divided in two structural classes: all-α structures, or all-β structures.
(0.02 MB EPS)

Text S1 Library of protein structures.
(0.04 MB PDF)

Video S1 A short movie of VAL60 trajectory during the biased molecular dynamics simulation.
(2.73 MB MPG)
