# Supplementary material for: Exploring the Universe of Protein Structures beyond the Protein Data Bank
Source: PLoS Comput Biol. 2010 Nov 4;6(11):e1000957. doi: 10.1371/journal.pcbi.1000957 (PMC2973819; doi:10.1371/journal.pcbi.1000957)
Supplement: Text S1 — Library of protein structures. (0.04 MB PDF) [file pcbi.1000957.s007.pdf]

Exploring the universe of protein structures beyond the Protein Data Bank

Pilar Cossio[1], Antonio Trovato[2,*], Fabio Pietrucci[1,3,*], Flavio Seno[2], Amos Maritan[2], Alessandro Laio[1]
1SISSA and CNR-INFM DEMOCRITOS, I-34157 Trieste, ITALY
2 Universitá degli Studi di Padova, and CNISM, Unitá di Padova, I-35131 Padova, Italy
3 Centre Européen de Calcul Atomique et Moléculaire (CECAM), Ecole Polytechnique Fédérale de Lausanne (EPFL) CH-1015 Lausanne, Switzerland
∗ **E-mail: antonio.trovato@pd.infn.it, fabio.pietrucci@epfl.ch**

Supplementary Information

Library of protein structures

For comparing the VAL60 structures with the existing folds in nature four different databases were considered: PDBSelect [1], TOP500 [2], CATH [3] and all the independent single domain proteins from the PDB (SD-PDB), as obtained searching the keywords: *protein*, *one chain*, and *no ligands* in the pdb repository. Each set was filtered by selecting only the proteins that had length L between 40 and 75 amino acids, had more than 30% of secondary structure, had no gaps and a gyration radius smaller than 15 Å. In order to find the independent structures, each set was further screened using the procedure described in Methods. After applying all these screens, the number of independent folds in PDBSelect, TOP500 and SD-PDB is smaller than 100. Instead the CATH set still contains 265 folds. For this reason, we choose CATH as our reference library of folds. The names of the CATH structures used in this work are given in table S1. We also performed tests aimed at verifying the capability of the VAL60 dataset of reproducing the databases PDBSelect, TOP500 and SD-PDB. The number of structures that are explored as a function of simulation time grows in all the cases in a manner that is qualitatively similar to what observed for the CATH database (data not shown).

References

1. Hobohm U, Scharf M, Schneider R, Sander C (1992) Selection of representative protein data sets. Protein Sci 1: 409-417.

2. Lovell S, Davis I, Adrendall W, de Bakker P, Word J, et al. (2003) Structure validation by C alpha geometry: phi,psi and C beta deviation. Proteins 50: 437-450.

3. Orengo C, Michie A, Jones S, Jones D, Swindells M, et al. (1997) CATH - a hierarchic classification of protein domain structures. Structure 5: 1093-1108.

1aipC03	1ayjA00	1b9wA01	1ck7A02	1djxB01	1e8gA04	1e8rA00	1ektA00	1el6A01	1extA02	1fbnA01	1fuqA03	1gaxA04	1ha8A00
1hicA00	1hw7A02	1jeqA05	1jroA03	1k8bA00	1lm8V01	1mpgA03	1poiA02	1tvkB03	1uglA00	1x9bA00	1xrsB01	1zwvA00	2bm0A03
2hbaA00	2jrrA01	2oyoA01	1c0mA02	1deeG00	1ptqA00	1gjzA00	1nh2D01	1amlA00	1atxA00	1bazA00	1bbgA00	1bgwA01	1bhpA00
1biaA03	1bzkA00	1c55A00	1ck7A03	1cvuA01	1e0eA00	1e0gA00	1e3oC02	1e8pA00	1ed7A00	1ehsA00	1ekeB02	1ep3B03	1eptA00
1erdA00	1f5tA02	1fbrA01	1fd3A00	1fs1A00	1g29102	1go3F02	1gp8A00	1h1jS00	1h59B00	1h5wB03	1h6wA01	1inpA01	1ji8A01
1k1vA00	1lkoA02	1m1eB01	1mbmB03	1ncsA00	1nd9A00	1olgA00	1pnkA02	1qhkA00	1qo0D02	1twfI01	1uhaA02	1vpuA00	1w4eA00
1y1bA00	2cxnA03	2hjqA01	2otkE00	5reqB03	1a5tA02	1aapA00	1aipE03	1au7A02	1b4aA01	1ci3M02	1d2dA00	1dd9A03	1dxsA00
1e4eA02	1efaA01	1gcyA02	1h3nA03	1h9eA00	1hywA00	1k3rA02	1ly2A02	1pg5B02	1qxfA00	1qypA00	1rk6A03	1sqgA03	1t50A00
1tkeA03	1u94A02	1vq0A02	1vq8W02	2bayE00	2gycX00	2j8gA03	1a76A02	1aiwA00	1b04A03	1b3qA04	1bl0A02	1bunB00	1bxyA00
1cseI00	1dkgA02	1dqaD01	1dtdB00	1eakA01	1eejA01	1eh9A02	1ex7A01	1f94A00	1fjrA01	1g19A02	1gccA00	1go3F01	1hz6B00
1i2tA00	1i9gA01	1inlC02	1j7mA00	1ji8A02	1jlcB04	1kfwA02	1koyA00	1kvdA00	1kxpD05	1on2A02	1pceA00	1r69A00	1rq6A00
1syxB00	1tkeA01	1ucsA00	1umqA00	1wqjI00	1xjhA00	1yuaA01	2cc6A00	2ecsA00	2fj8A01	2gpfA01	2hg7A00	2jr6A01	2jrmA00
2nn4A00	3bulA04	1b3aA00	1b69A00	1b8tA01	1bbyA00	1bcoA02	1brwA03	1c7sA04	1c7vA00	1ccwB02	1d2nA02	1dvpA02	1ehiA03
1elvA03	1hp8A00	1j2zA02	1jajA02	1k0rA04	1khcA02	1ky9B04	1l6hA00	1lq7A00	1mhyG01	1mntA00	1mpxA02	1musA01	1qzpA00
1rrzA00	1tvfA02	1uxyA02	1v0eA01	1xakA00	1xccA02	2derA02	2hjjA00	2jn4A00	2nllA00	2proC01	1nh8A03	1ib8A02	1c4qA00
1cqqA01	1i6uA01	1qyrA02	1a79A02	1a9xA03	1apjA00	1au7A01	1b22A00	1b6rA01	1cfaA00	1cktA00	1dzfA02	1e8oA00	1eiaA02
1eijA00	1f3mA00	1fjgR00	1g8lA04	1gh9A00	1hc7B03	1ic8A02	1iq8A02	1iq8A03	1je3A01	1jw2A00	1kgqA01	1ku1A01	1mmsB00
1o54A01	1os6A00	1pgxA00	1pkpA01	1qsaA02	1r8eA02	1tolA02	1uj8A00	1vajA02	1vqqA03	1wj2A00	1zjaA02	2g2uB01	2jovA01
2nocA01	1aw0A00	1cidA02	1fx0A01	2px6A02	1dj7B00	2a3dA00	1a62A02	1axnA01	1b0xA00	1b24A02	1cpyA02	1fjgM01	

Table 1. List of the names for CATH folds used in this work.
